# Antarctic moss fairy rings serve as reservoirs for plant growth-promoting bacteria

**DOI:** 10.1186/s12870-026-08127-3

**Published:** 2026-01-20

**Authors:** Huiwon Choi, Yelim Lee, Hongshi Jin, Jihyeon Yu, Hyoungseok Lee, Doil Choi, Jungeun Lee

**Affiliations:** 1 Division of Life Sciences, Korea Polar Research Institute, Incheon, South Korea; 2 Department of Agriculture, Forestry and Bioresources, Seoul National University, Seoul, Korea; 3 Department of Biological Sciences, Seoul National University, Seoul, Korea; 4 Polar Science, University of Science and Technology, Incheon, South Korea

**Keywords:** Antarctic moss, Sanionia uncinata, Fairy rings, Plant growth-promoting bacteria, Sphingomonas, Rhizobium

## Abstract

**Background:**

Antarctic terrestrial vegetation and microbial communities are undergoing climate-driven changes, which in some cases have been linked to the emergence of plant diseases. Ring-shaped plant disease structures, known as fairy rings (FRs), have been observed in moss fields dominated by *Sanionia uncinata*. Previous studies suggest that FRs promote vegetation development through interactions with bacterial communities. This raises the possibility that the bacterial communities in Antarctic FRs may also have ecologically significant functions. To address this, we analyzed the bacterial consortia of *S. uncinata* FRs, isolated key bacterial strains, and evaluated their plant growth-promoting abilities through physiological experiments.

**Results:**

Amplicon sequence variable (ASV)-based bacterial community analysis revealed that *Sphingomonadales* were most abundant in tissues forming FRs compared to healthy *S. uncinata* tissues. Microbial isolation yielded 28 strains belonging to the genera *Sphingomonas*, *Rhizobium*, and *Tardiphaga*, which were significantly enriched in FRs. Among them, *Sphingomonas* spp. and *Rhizobium* spp. exhibited strong activities in cellulose degradation, phosphate solubilization, and production of indole-3-acetic acid, a plant hormone. The effects of these strains on promoting increases in plant biomass and root development were verified through physiological experiments using *Arabidopsis thaliana* seedlings.

**Conclusions:**

In this study, ASV-based metabarcoding analysis and physiological assays with bacterial isolates demonstrated that the dominant bacteria in Antarctic FRs have the potential to promote plant growth in Antarctica. These findings contribute to the understanding of the dynamic development of the Antarctic terrestrial ecosystems, where plants, bacteria, and fungi interact closely under the influence of climate change.

**Supplementary Information:**

The online version contains supplementary material available at 10.1186/s12870-026-08127-3.

## Background

Antarctic terrestrial ecosystems are among the most extreme environments for biological colonization and are characterized by harsh conditions that limit the adaptability of life [1, 2]. In this region, mosses and lichens drive complex biogeochemical changes, enabling dynamic ecosystem development and setting conditions for subsequent biological colonization [3].

Mosses, which are adapted to the harsh climate of Antarctica, serve as critical primary colonizers because of their exceptional physiological resilience [4]. These mosses exhibit remarkable tolerance to extreme temperature fluctuations and limited water availability. They progressively alter the substrate conditions, forming microenvironments that support further biological colonization [4–6]. *Sanionia uncinata*, a dominant moss species in maritime Antarctica, has mechanisms that allow it to withstand harsh environments that limit plant development [1, 7]. These characteristics enabled the Antarctic moss *S. uncinata* to thrive in the Antarctic terrestrial ecosystem and serve as the foundation for various ecological phenomena to occur.

In Antarctic moss carpets, a phenomenon known as the “fairy ring” has been frequently observed and is characterized by fungal infections that cause concentric rings of browning and moss mortality [8–13]. Fairy rings (FRs), which are traditionally understood as areas represented by circular rings in vegetation, constitute unique ecological microhabitats. In various ecosystems, from grasslands to Antarctic moss carpets, FRs have historically been studied for their pathogenicity and the saprophytic roles of associated fungi [9, 11–16].

However, recent studies have recognized FRs as complex and diverse microenvironments. Studies in Mediterranean grasslands have reshaped our understanding of FRs, revealing them as dynamic ecological structures that increase microbial diversity and support community interactions rather than mere pathological zones [17]. Such insights are particularly relevant to Antarctic ecosystems, where glacial retreat creates nascent landscapes that require both rapid and strategic biological colonization. In Antarctica’s resource-limited environments, microbial consortia within FRs may serve as vital ecological mediators that promote nutrient mobilization, create microhabitats, and support early ecosystem reconstruction glacial retreat [18]. These dynamic microhabitats suggest the importance of investigating their role in facilitating plant adaptation and succession. The bacteria and fungi within FRs engage in synergistic interactions that increase the complexity of Antarctic ecosystem processes, revealing a multifaceted ecological process [19, 20]. Thus, understanding the microbial communities within FRs that support the establishment of subsequent plant communities is essential.

Plant growth-promoting bacteria (PGPB) are key components that increase plant adaptation to harsh environments [21–23]. Through complex biochemical pathways, PGPB provide critical ecosystem services, including enzymatic nutrient solubilization, asymbiotic nitrogen fixation, the biosynthesis of phytohormones such as indole-3-acetic acid (IAA), and antagonistic biocontrol responses against phytopathogens *via* siderophore production, hydrogen cyanide generation, and antimicrobial metabolite secretion [24, 25]. Previous studies have reported an increase in the abundance of beneficial microorganisms that may assist in mycorrhizal formation in FR, and these have also been classified as PGPR with plant growth-promoting functions [17, 26–28]. Although FRs and their bacterial communities have been well studied in grassland ecosystems [17, 29, 30], the bacterial diversity and ecological roles of *S. uncinata* FRs in Antarctic ecosystems remain poorly understood. Moss-associated microbial communities play important roles in high-latitude ecosystems, contributing to functions such as soil organic matter decomposition and nitrogen fixation [31–33]. Given their ecological importance, the taxonomic composition of microbial communities within moss FRs has been characterized [34, 35]. However, the functional potential of these bacterial consortia in promoting plant growth remains largely unknown. Previous studies on FRs in grassland ecosystems caused by the fungus *Leucocalocybe mongolica* have provided valuable insights into plant growth-promoting bacterial strains, such as *Bacillus pumilus* [36]. However, these studies have not fully explored the distribution or dominance of these bacteria within the microbial community.

To overcome the limitations of previous studies, this study investigated bacterial communities in *S. uncinata* moss FRs in Antarctic ecosystems, analyzed their community structure, isolated dominant strains, and evaluated PGPB functions. We validated their plant growth-promoting potential using model plant systems to explore whether these traits might contribute to ecological processes that occur during early vegetative development under extreme Antarctic conditions. Although further evidence is needed, our integrated approach suggests that these microbial consortia may play supportive roles within localized Antarctic terrestrial ecosystems.

## Methods

### Site description and collection of *S. uncinata* with FRs

In January 2021, a survey of FR-influenced moss samples (*Sanionia uncinata*) was conducted at a coastal site in the vicinity of King Sejong Station, King George Island, located in maritime Antarctica (62°13′22″S, 58°47′18″W). Sampling was carried out within an approximately 20 m × 20 m area where *S. uncinata* carpet mats are well developed and where FRs (FR) occur frequently. From this site, visibly symptomatic FR tissues were collected from at least three independent biological replicates to capture variability across sampling points within the site. Asymptomatic proximal zone (PL) samples were collected in the same manner from the same site, and healthy zone (HL) samples were obtained in triplicate from mats with no visible FRs in the vicinity of the station. While this sampling design was confined to a local area, the sampling sites were selected to represent *S. uncinata*-dominated mats, ensuring optimal samples were obtained for detecting the effects of FRs.

For metabarcoding analysis, samples for DNA extraction were preserved in 80% ethanol immediately after collection. The samples intended for bacterial isolation were suspended in 20% glycerol and transported in a frozen state until processing in the laboratory.

The population of *S. uncinata* at the study site had previously been formally identified by the bryophyte taxonomist Dr. Young-Jun Yoon. Voucher specimens (ID: KOPRI-MO00156, MO00157, MO00159) from this population have been preserved in the Korea Polar Research Institute Herbarium (https://kvh.kopri.re.kr). Species identity was further confirmed by sequencing analyses, including 16S rRNA and ITS metabarcoding, which revealed that the majority of plant-derived sequences in the community were assigned to *S. uncinata*.

### Metabarcoding analysis on the basis of the amplicon sequence variable (ASV)

The samples for DNA extraction were frozen in liquid nitrogen and then ground using a pestle. Holobiont DNA for metabarcoding was extracted from the ground tissue using the DNeasy Plant Mini Kit (QIAGEN). Sequencing libraries were prepared following the Illumina 16S metabarcoding sequencing library preparation protocol to amplify two targets: the V3–V4 region of the 16S rRNA gene and the ITS2 region of internal transcribed spacer (ITS) region. Subsequently, second PCR was performed for final library construction with Nextera XT Indexed Primer. The final libraries were quantified using qPCR according to the qPCR Quantification Protocol Guide (KAPA Library Quantification kits for Illumina Sequencing platforms) and qualified using the TapeStation D1000 ScreenTape (Agilent Technologies, Waldbronn, Germany). The paired-end (2 × 300 bp) sequencing was performed using the MiSeq™ platform (Illumina, San Diego, USA).

The raw sequencing data were processed using QIIME2 (version 2024.10) [37]. Amplicon sequencing targeting the 16S rRNA gene and ITS region of the rRNA gene was performed, and the raw reads were processed using DADA2 [38] for denoising and construction of amplicon sequence variants (ASVs). Prior to denoising, the quality profiles of the demultiplexed reads were determined. Low-quality regions were then removed by trimming the first 15 bp of both forward and reverse reads and truncating them at 260 bp (forward) and 200 bp (reverse). This process selected reads suitable for analysis, and at least 38,000 reads per sample were subsequently used for analysis.

Taxonomic classification of 16S rRNA gene ASVs was conducted with the BLAST algorithm against the SILVA SSU database (version 138.2) [39], with a minimum identity threshold of 99%, e-value cutoff of 0.001, and top-hit assignment criteria. The ITS region was analyzed *via* the UNITE classifiers for QIIME (version 10.0) [40]^,^ and its cutoff was the same as that used for 16S rRNA analysis. ASVs that were classified as ‘Unassigned’, ‘Mitochondria’ or ‘Chloroplast’ origin were filtered out from downstream analyses to focus on bacterial community structure. In the fungal community, only ASVs categorized as ‘Unassigned’ were excluded from the analysis. To compare taxon distributions, analyses were performed based on the relative abundance of each taxon within individual samples to minimize the bias from varying sequencing depth across samples.

Microbial community diversity was analyzed using the ‘diversity core-metrics-phylogenetic’ command after obtaining a phylogenetic tree with the ‘phylogeny align-to-tree-mafft-fasttree’ command in QIIME2. The alpha diversity of each microbial group was evaluated on the basis of the Shannon index and the Faith-PD, and the beta diversity was plotted as a PCoA plot by calculating the Bray‒Curtis index. Normalization in diversity analysis was performed by rarefying to the sample with the lowest read count. The statistical significance of alpha diversity was assessed using the Kruskal–Wallis test, followed by post–hoc multiple comparisons in GraphPad Prism 10 (version 10.2.3; https://www.graphpad.com/) based on the diversity indexes identified across the three sample types (3 replicates per sample type). Beta diversity was also statistically significant as confirmed by PERMANOVA. A permutation test was performed 999 times using the adonis2 function from the vegan R package. The significance level of the diversity analysis was p-value < 0.05. The results of the diversity analysis were visualized using RStudio (version 4.3.0) [41].

### Isolation, identification and ASV sequence match of endophytic bacteria

The endophytic bacteria used in this study were isolated from a sample of FR *S. uncinata*. First, the moss sample was washed with sterile water three times and then placed into a 1.5 mL microtube containing 1 mL of NaCl (0.85%) solution. The mixture was ground, and the extract mixture was placed on 1/5 potato dextrose agar (PDA) medium (DB Difco, 213400). Individual colonies were isolated after 2 weeks of incubation in a 16°C dark chamber.

For identification of the isolated bacterial strain, approximately 1,400 bp of the 16S rRNA gene sequence of the isolate was amplified by PCR using the universal bacterial primers 27 F (5′-AGAGTTTGATCCTGGCTGGCTCAG-3′) and 1492R (5′-TACGGCTACCTTGTTACGACTT-3′). Amplified 1S rRNA sequences were aligned to the best matching species with high identity *via* BLASTn in the NCBI-GenBank database (https://blast.ncbi.nlm.nih.gov). To select only the ASVs that matched the isolated strains from the ASV pool obtained from the sample, the ASV sequences were matched to the 16S rRNA sequences of the isolated bacteria. Using the 410 ~ 415 bp ASV sequence as a reference, the 1,400 bp 16S rRNA of the isolated strains was aligned using Geneious Prime (version 2024.0.5; https://www.geneious.com). Only when the V3–V4 region of the isolated strain’s 16S rRNA sequence perfectly matched the ASV sequence with 100% pairwise identity was the strain considered a ‘matched FRB strain’. The differences between FR, PL, and HL were tested for statistical significance using the Kruskal‒Wallis test based on the matched ASVs as implemented in GraphPad Prism 10. The significance level was p-value < 0.05.

### Phylogenetic analysis of bacterial isolates

Phylogenetic analysis was performed on FRB strains belonging to the genera: *Sphingomonas*, *Rhizobium*, and *Tardiphaga* using MEGA (version 11) [42]. Neighbor-Joining phylogenetic tree was constructed using 16S rRNA sequences, including the strains matched in the BLAST results of the isolated strains and the type species of the respective genera. The substitution model used for tree construction was the Maximum Composite Likelihood model, and bootstrap replication was performed 100 times.

### Bacterial pathogenicity test using *Nicotiana benthamiana* leaves

Endophytic bacteria were cultured in 1/5 potato dextrose broth (PDB) (DB Difco, 254920) for 2 weeks at 16°C, and *Pseudomonas syringae* pv. tomato strain DC3000 (*Pst* DC3000) [43] cultured in King’s B media at 28 °C for 2 days were prepared. The bacterial cultures were subsequently centrifuged at 25 °C and 4000 rpm for 10 min, after which the supernatant was discarded. The pellet was subsequently resuspended in sterilized distilled water to produce a bacterial suspension for infiltration. The concentration of *Pst* DC3000 used for infiltration was based on crop inoculation cases [44], and the OD_600_ of the isolated endophytic bacteria was standardized to 0.4. For endophytic bacteria of the same genus, a mixture was prepared. A 1 mL needleless syringe was used to infiltrate the bacterial suspension onto the abaxial side of five-week-old *N. benthamiana* leaves. The plants were then incubated at 22 °C for 21 days under observation.

### Bacterial pathogenicity test using potato tubers

Endophytic FRB cultured in PDB at 16°C for 2 weeks and a positive control *Pectobacterium carotovorum* (KACC 16999) [45, 46] cultured in R2A liquid medium at 25 °C for one day were centrifuged at 25 °C and 4000 rpm for 10 min to remove the supernatant. The pellet was diluted with 0.9% NaCl to prepare a bacterial suspension. A mixture of endophytic bacteria of the same genus was prepared. The concentration of endophytic bacteria was the same as that used in the infiltration experiment, and the OD_600_ was adjusted to 0.1. The potatoes were washed with running water to remove the soil, soaked in 10% ethanol for 5 min, rinsed twice with sterile distilled water and dried overnight. The dried potatoes were sliced to a thickness of 1 cm and placed in a square dish (125 × 125 × 20 mm) with filter paper soaked in sterile distilled water. Wells (4.5 × 4.5 mm) were made in the center of each potato slice, and 30 µl of bacterial suspension was added. The dish was then incubated at 16°C for 7 days and observed.

### Enzyme production activity assay

Cellulase production was determined on carboxymethyl cellulose (CMC) agar. CMC agar contained 2 g of carboxymethylcellulose sodium salt, 0.5 g of yeast extract, 0.5 g of proteose peptone No. 3, 0.5 g of casamino acid, 0.5 g of dextrose, 0.5 g of soluble starch, 0.3 g of sodium pyruvate, 0.3 g of K_2_HPO_4_, 0.05 g of MgSO₄, 15 g of agar, and 1 L of distilled water. The final pH was adjusted to 7.2, and the mixture was autoclaved at 121 °C for 15 min. Bacteria cultured in 1/5 PDB liquid medium for 2 weeks were spotted on CMC agar in 10 µl aliquots and incubated at 16°C for 7 days. After 7 days, 4 mL of 0.1% Congo red dye was added to the CMC agar and incubated for 10 min. Then, 6–10 mL of 1 M NaCl was added to each plate, and the colonies were removed with a loop and washed four times. The transparent zones on the CMC agar plates were quantified *via* ImageJ. The images were converted to grayscale (8 bits) to minimize color interference. The mean gray values were measured at three points: the clearing zone, the intact agar medium, and the black background. The transparency index was calculated as follows:

$$Transparency\:Index\:\left(\%\right)={\frac{({G}_{clear}-{G}_{medium})}{{(G}_{medium}-{G}_{background})}\times\:100}_{\:}$$

where $\:{G}_{clear},\:{G}_{medium},\:{G}_{background}$ represent the mean gray values of the clearing zone, the medium, and the background, respectively. Strains with a transparency index greater than 3% were considered to have cellulase-producing activity.

### Phosphate solubilization activity assay

The phosphate solubilization activity of the endophytic bacteria was assessed by culturing on Pikovskaya (PVK) agar plates. The medium consisted of 10 g of glucose, 2.5 g of tricalcium phosphate Ca_3_(PO_4_)_2,_ 0.5 g of ammonium sulfate (NH_4_)_2_SO_4_, 0.2 g of NaCl, 0.2 g of KCl, 0.1 g of MgSO_4_⋅7H_2_O, 2 mg of MnSO_4_, 2 mg of FeSO_4_⋅7H_2_O, 0.5 g of yeast extract, and 15 g of agar in 1 L of distilled water, with the pH adjusted to 7.0. Colonies of bacterial isolates were inoculated into the center of PVK agar media and incubated at 16°C for 7 days. A transparent halo zone around the colony of the inoculated bacterial strain indicates positive phosphate solubilization. Phosphate solubilization activity were quantified by calculating the clearing ratio of the transparent halo diameter (D, cm) to the colony diameter (d, cm), expressed as D/d [47, 48]. It was evaluated as having activity when D/d > 0.

### Siderophore production activity assay

Siderophore production was determined on chrome azurol S (CAS) agar. CAS agar requires several reagents, including blue dye, minimal media 9 (MM9) salt solution stock, 20% glucose stock, NaOH stock, and casamino acid solution. 1) To make blue dye, first dissolve 60.5 mg of CAS (Fluka Chemicals) in 50 ml of distilled water to make Solution (1) Then, 2.7 mg of FeCl_3_.6H_2_O was dissolved in 10 ml of 10 mM HCl to make Solution (2) Next, 72.9 mg of HDTMA was dissolved in 40 ml of distilled water to make Solution (3) Solution 1 and Solution 2 were combined and mixed well before Solution 3 was added. The resulting mixture turned blue in color. 2) The minimal media 9 (MM9) salt solution stock contained 64 g of Na_2_HPO_4_⋅7H2O, 15 g of KH_2_PO_4_, 2.5 g of NaCl, 5 g of NH_4_Cl and 1 L of distilled water. 3) A 20% glucose stock mixture is made by dissolving 20 g of glucose in 100 ml of distilled water. 4) The NaOH stock solution is made by dissolving 25 g of NaOH in 150 ml of distilled water. The pH was adjusted to 12. 5) To prepare the Casamino Acid Solution, dissolve 3 g of Casamino acid in 27 ml of distilled water. To remove any trace iron, 27 ml of 3% 8-hydroxyquinoline was extracted in chloroform at 4 °C overnight. The solution was sterilized to ensure its purity. Siderophore production ability was quantified in the same way as the method of quantifying phosphate solubilization activity. It was evaluated as having activity when D/d > 0.

### IAA production assay

A single colony of the strain was cultured in 10 ml of R2A media supplemented with 2.5 mM tryptophan at 25 °C for 3 days, and the OD_600_ was measured. Then, the samples were centrifuged at 4 °C and 10,000 rpm for 10 min, and only the supernatant was isolated. The supernatant was mixed with Salkowski reagent (12 g/L FeCl_3_, 7.9 mol/L H_2_SO_4_) at a ratio of 1:1 (v/v), incubated for 30 min at room temperature in the dark, and the absorbance was measured at 530 nm. The measured absorbance was quantified on the basis of an OD_600_ of 0.4. 3-Indoleacetic acid (Sigma, 87-51-4) was used to construct a standard curve for quantification.

### Plant growth promotion test

*Arabidopsis thaliana* seeds were surface sterilized with 70% ethanol and 0.05% Tween 20 solution for 20 min, followed by two rinses with 100% ethanol on a clean bench. After being air-dried on filter paper for 30 min, the seeds were sown in rows on 1/2 MS agar plates (2.15 g/L MS salts, 15 g/L sucrose, and 8 g/L phyto agar, pH 5.7) and subjected to short cold treatment at 4 °C in the dark for three days. The plates were then transferred to a growth chamber (22 °C, 16 h light/8 h dark) for six days.

For bacterial preparation, single colonies of strains were cultured in R2A media (3.15 g/L R2A broth, pH 7.0), harvested by centrifugation (3,000 rpm, 10 min), and resuspended in fresh medium to a final OD_600_ of ~ 0.4. *Pseudomonas putida* (KACC 10266) was used as a plant growth-promoting (PGP) control strain [49, 50], while sterile R2A broth and autoclaved *P. putida* suspensions served as negative controls.

For co-culture, the upper part of the 1/2 MS plate was cut off and discarded so that the plants could grow without attaching to the surface of the medium when transplanted. To ensure enough bacterial growth on the 1/2 MS plate, 10 µl of bacterial suspension was streaked on the bottom of the plate. Since growth patterns differed among strains, the inoculation dates were adjusted accordingly. The plates were incubated vertically at 22 °C under 16 h light/8 h dark conditions for 11 days. Plant growth was assessed by measuring the fresh weight and number of secondary roots of individual plants. For each treatment, more than 20 individual plants per group (*n* > 20) were analyzed across triplicates. Statistical significance was verified with one-way ANOVA test when comparing fresh weight and the number of secondary roots between groups. Post-hoc multiple comparisons were performed in GraphPad Prism 10 to determine statistically significant differences between groups. When comparing growth rates between groups, the percentage of increase or decrease was described relative to the average of the measured values.

## Results

### Microbial community structure analysis for *S. uncinata* FR

**Fig. 1:**
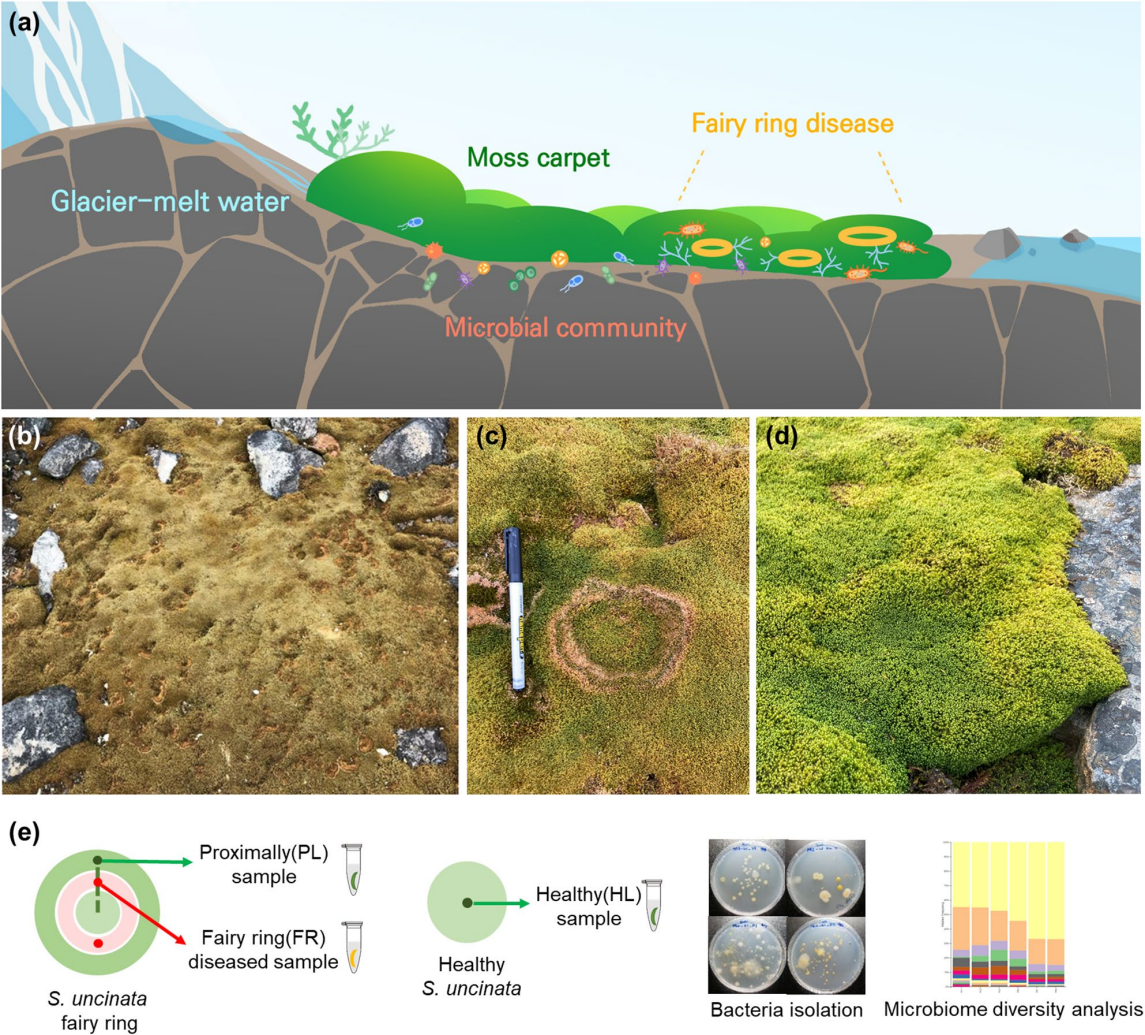
Sampling sites and overview of experimental workflow. **a** Schematic diagram of the sampling site on the Barton Peninsula, King George Island, Antarctica, depicting the moss carpets and fairy rings in Antarctic vegetation. **b**,** c** Field photographs of *S. uncinata* mats with visible fairy rings. **d** Representative photograph of a healthy *S. uncinata* mat (HL). **e** Overview of sample groups (FR, proximal PL and HL) and schematic diagram illustrating the isolation of bacterial strains and taxonomic microbiome analysis based on ASV data

The study site, Barton Peninsula on King George Island, hosts abundant *S. uncinata* moss growing in moist areas near glacier-meltwater streams. The climate is harsh, with daily mean air temperatures ranging from 0 °C to 5 °C during summer [51]. FRs, characterized by concentric vegetation patterns, are very common features within these moss carpets (Fig. 1a). We collected three types of *S. uncinata* samples, FR, PL, and HL, to compare the microbial community distributions between healthy *S. uncinata* and *S. uncinata* exhibiting a FR phenotype (Figs. 1b-e) based on an ASV-based analysis. Sequencing data for 16S rRNA and ITS were obtained in triplicate per sample type, with 346 ASVs obtained for 16S rRNA and 287 ASVs obtained for ITS (Supplementary Table S1-2).

**Fig. 2:**
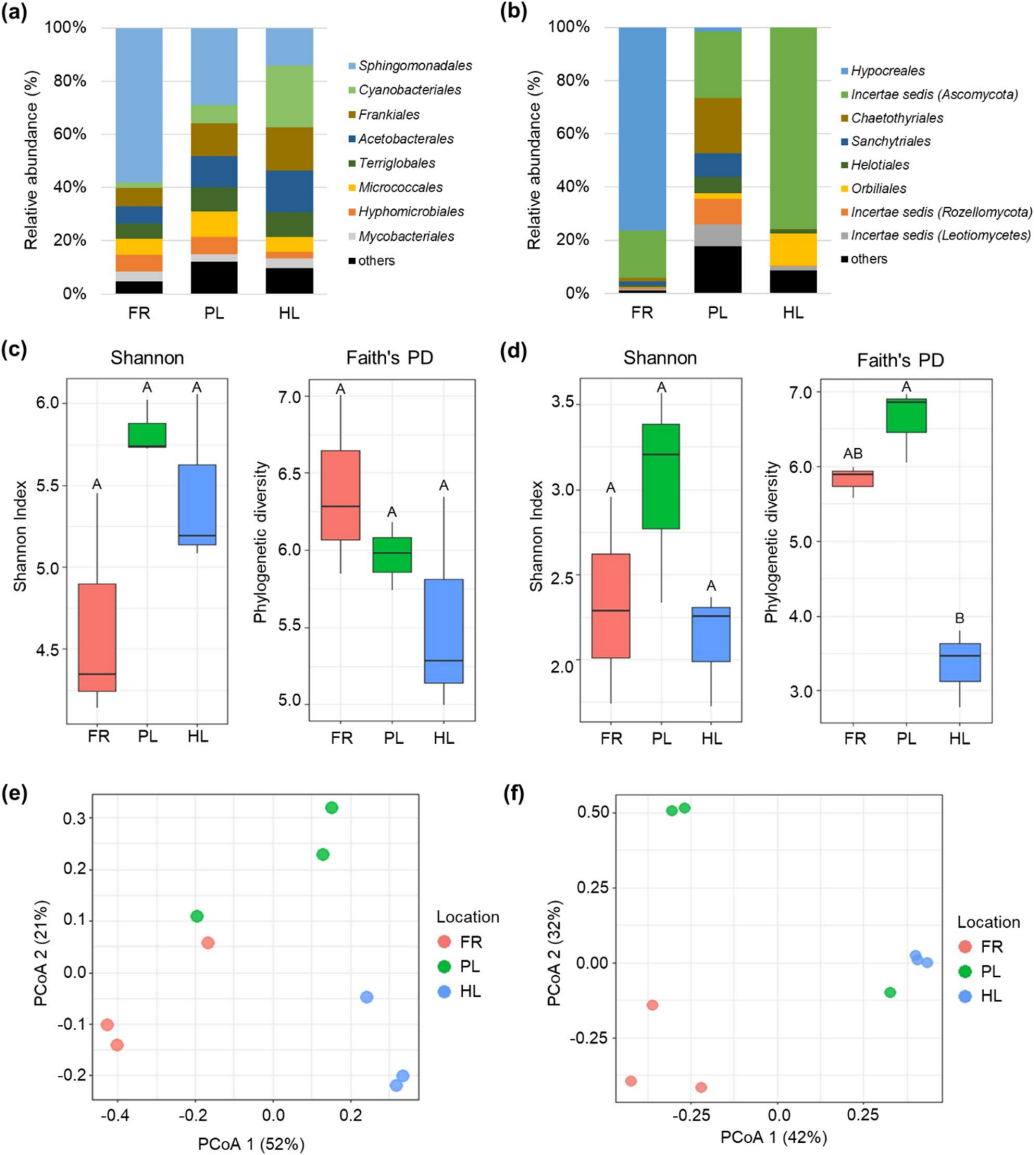
Microbial community composition and diversity in *S. uncinata* fairy rings. Relative abundance of (**a**) bacterial and (**b**) fungal taxa at the order level. Alpha diversity analyses of (**c**) bacterial and (**d**) fungal communities, and beta diversity analyses of (**e**) bacterial and (**f**) fungal communities, comparing FR, PL, and HL samples

At the order level, *Sphingomonadales* accounted for an average of 58.2% of the bacteria in the FR samples, decreasing to 28.9% in the PL samples and 14.1% in the HL samples (Fig. 2a). Overall, *Pseudomonadota* accounted for more than 70% of the FR bacterial community. This dominance was strongest in the FR samples and declined in the PL (51.6%) and HL (34.7%) samples. These results indicate that the FR region of *S. uncinata* is characterized by significant enrichment of *Pseudomonadota*, driven mainly by *Sphingomonadales*.

At the order level of fungal community, 76.5% of the fungi were classified as *Hypocreales*, but only 1.9% were identified in the PL sample sampled in the immediate vicinity, and none were identified in the HL sample (Fig. 2b). This *Hypocreales* was identified at the species level as *Psychronectria hyperantarctica*. When including the unclassified clade ‘*Incertae_sedis’* within the *Ascomycota* phylum, *Ascomycota* accounted for more than 95% of FR samples. Compared to other locations, fungi classified as *Chaetothyriales*, *Sanchytriales*, *Helotiales*, *Incertae_sedis* (*Rozellomycota* and *Leotiomycetes*) accounted for a significant proportion in the PL samples. In particular, *Cladophialophora* dominated within the *Chaetothyriales*. In the HL samples, *Incertae_sedis* (*Ascomycota*) dominated at 75.8%.

Alpha diversity of bacterial communities showed no statistically significant differences in both Shannon index and Faith’s PD values (Fig. 2c). The Faith’ PD index of bacterial communities showed a tendency for diversity to decrease as it approached the HL sample. In contrast, Faith’s PD values were significantly lower in HL samples in fungal group (Fig. 2d). Beta diversity analysis using principal coordinate analysis (PCoA) demonstrated separation of the FR, PL, and HL sample groups (Figs. 2e, f) and these separations were statistically significant (p-value < 0.05).

### Taxonomic profiling and FRB isolation

To characterize the bacterial community associated with *S. uncinata* FRs, we first performed high-resolution ASV-based taxonomic profiling, which revealed significant enrichment of specific genera within FR (FR) samples (Table 1). Specifically, ASVs assigned to the genus *Sphingomonas* (ASV1 and ASV3) presented a relative abundance of approximately 42.8% in the FR samples, which was substantially greater than that in the proximal (PL) and healthy (HL) samples. The abundance of ASV belonging to the genus Rhizobium (ASV14) increased, accounting for approximately 2.5% of the FR microbial community. The genus *Tardiphaga* (ASV62) was not detected in any of the other samples but was found only in the FR sample.

**Table 1:** The FRB strains presented multiple matches with high-ranking ASV

ASV ID	Relative abundance (%)									*p* value	Matched FRB strain	Organism
	FR1	FR2	FR3	PL1	PL2	PL3	HL1	HL2	HL3			
ASV1	43.4	17.0	43.0	3.8	1.8	3.4	0.2	0.0	1.5	0.007*	FRB18	*Sphingomonas* sp.
ASV3	7.0	9.6	4.2	10.8	6.0	7.3	2.8	1.8	2.7	0.042*	FRB19	*Sphingomonas* sp.
ASV14	2.9	1.9	2.7	2.7	2.3	2.0	0.5	0.4	0.9	0.046*	FRB14	*Rhizobium* sp.
											FRB15	*Rhizobium* sp.
											FRB16	*Rhizobium* sp.
											FRB17	*Rhizobium* sp.
ASV33	1.8	0.8	1.7	1.3	0.0	0.0	0.0	0.0	0.0	0.071	FRB2	*Rhodococcus* sp.
ASV74	0.9	0.0	0.6	0.0	0.0	0.0	0.0	0.0	0.0	0.250	FRB13	*Tardiphaga* sp.
ASV16	0.6	1.0	0.5	0.5	3.1	2.6	1.0	1.2	2.5	0.607	FRB6	*Lacisediminihabitans* sp.
ASV12	0.6	0.9	0.5	1.0	2.5	3.0	1.3	1.7	4.5	0.357	FRB7	*Nakamurella* sp.

* Indicates statistical significance at *p* < 0.05

To validate and functionally investigate the FR-enriched, dominant taxa revealed by high-resolution mapping, we performed culture-based isolation of bacterial strains from *S*. *uncinata* exhibiting FR disease. As a result, we obtained 28 strains belonging to 4 phyla and 18 genera, designated as FR bacteria (FRB) (Table 2). The 16S rRNA sequences of 10 FRB strains were identified as exactly matching the ASVs detected in the FR, PL, and HL samples. These included genera enriched in FRs, such as *Sphingomonas* and *Rhizobium* (Table 1).

**Table 2:** Twenty-eight FRB strains isolated from FR-affected *S. uncinata*

Name	Strain accession No.	Phylum	Order	Scientific name	Matched accession No.	Identities
FRB1	PX599208	*Actinomycetota*	*Corynebacteriales*	*Rhodococcus gannanensis*	NR_152643.1	95.34%
FRB2	PX599209	*Actinomycetota*	*Corynebacteriales*	*Rhodococcus gannanensis*	NR_152643.1	95.91%
FRB3	PX599210	*Actinomycetota*	*Corynebacteriales*	*Mycobacterium aquiterrae*	NR_158076.1	98.42%
FRB4	PX599211	*Actinomycetota*	*Corynebacteriales*	*Mycolicibacterium hippocampi*	NR_134094.1	97.99%
FRB5	PX599212	*Actinomycetota*	*Corynebacteriales*	*Mycolicibacterium rhodesiae*	NR_025529.1	97.85%
FRB6	PX599213	*Actinomycetota*	*Micrococcales*	*Lacisediminihabitans profunda*	NR_169497.1	98.21%
FRB7	PX599214	*Actinomycetota*	*Nakamurellales*	*Nakamurella panacisegetis*	NR_108869.1	99.43%
FRB8	PX599215	*Bacteroidota*	*Sphingobacteriales*	*Mucilaginibacter phyllosphaerae*	NR_152043.1	97.99%
FRB9	PX599216	*Bacillota*	*Bacilales*	*Priestia aryabhattai*	NR_115953.1	100.00%
FRB10	PX599217	*Pseudomonadota*	*Hyphomicrobiales*	*Lichenibacterium ramalinae*	NR_169335.1	98.16%
FRB11	PX599218	*Pseudomonadota*	*Hyphomicrobiales*	*Methylobacterium goesingense*	NR_115219.1	99.34%
FRB12	PX599219	*Pseudomonadota*	*Hyphomicrobiales*	*Methylobacterium adhaesivum*	NR_125492.1	99.19%
FRB13	PX599220	*Pseudomonadota*	*Hyphomicrobiales*	*Tardiphaga robiniae*	NR_117178.1	98.46%
FRB14	PX599221	*Pseudomonadota*	*Hyphomicrobiales*	*Rhizobium binae*	NR_137242.1	98.53%
FRB15	PX599222	*Pseudomonadota*	*Hyphomicrobiales*	*Rhizobium aegyptiacum*	NR_137399.1	98.53%
FRB16	PX599223	*Pseudomonadota*	*Hyphomicrobiales*	*Rhizobium tibeticum*	NR_116254.1	98.45%
FRB17	PX599224	*Pseudomonadota*	*Hyphomicrobiales*	*Rhizobium tibeticum*	NR_116254.1	98.53%
FRB18	PX599225	*Pseudomonadota*	*Sphingomonadales*	*Sphingomonas aerolata*	NR_042130.1	98.83%
FRB19	PX599226	*Pseudomonadota*	*Sphingomonadales*	*Sphingomonas glacialis*	NR_117270.1	99.85%
FRB20	PX599227	*Pseudomonadota*	*Burkholderiales*	*Caballeronia sordidicola*	NR_104563.1	98.93%
FRB21	PX599228	*Pseudomonadota*	*Burkholderiales*	*Variovorax boronicumulans*	NR_114214.1	99.57%
FRB22	PX599229	*Pseudomonadota*	*Burkholderiales*	*Rugamonas rubra*	NR_104915.1	98.86%
FRB23	PX599230	*Pseudomonadota*	*Pseudomonadales*	*Pseudomonas fluorescens*	NR_115715.1	99.72%
FRB24	PX599231	*Pseudomonadota*	*Pseudomonadales*	*Pseudomonas frederiksbergensis*	NR_117177.1	99.86%
FRB27	PX599232	*Pseudomonadota*	*Pseudomonadales*	*Pseudomonas bohemica*	NR_159101.1	98.02%
FRB29	PX599233	*Pseudomonadota*	*Pseudomonadales*	*Pseudomonas brenneri*	NR_025103.1	99.72%
FRB32	PX599234	*Pseudomonadota*	*Xanthomonadales*	*Rhodanobacter umsongensis*	NR_108435.1	97.89%
FRB33	PX599235	*Pseudomonadota*	*Rhodospirillales*	*Acidiphilium cryptum*	NR_025851.1	93.70%

Only strains with 100% sequence identity between the ASV and 16S rRNA gene of the FRB strain were classified as ‘matched FRB strain’. ASVs with at least 100 reads in more than one sample were considered for matching to FRB strains. Statistical difference in ASV distribution among FR, PL and HL samples were assessed using the Kruskal‒Wallis test, with p-value < 0.05 marked with an asterisk.

### FRBs have potential beneficial functions for plant growth

To identify the plant interaction functions of the isolated FRBs, we performed plant pathogenicity and PGP tests. Pathogenicity tests on *N. benthamiana* leaves and potato tubers showed that plants inoculated with FRB exhibited no or only mild symptoms compared with those of the controls (Fig. S1).

**Fig. 3:**
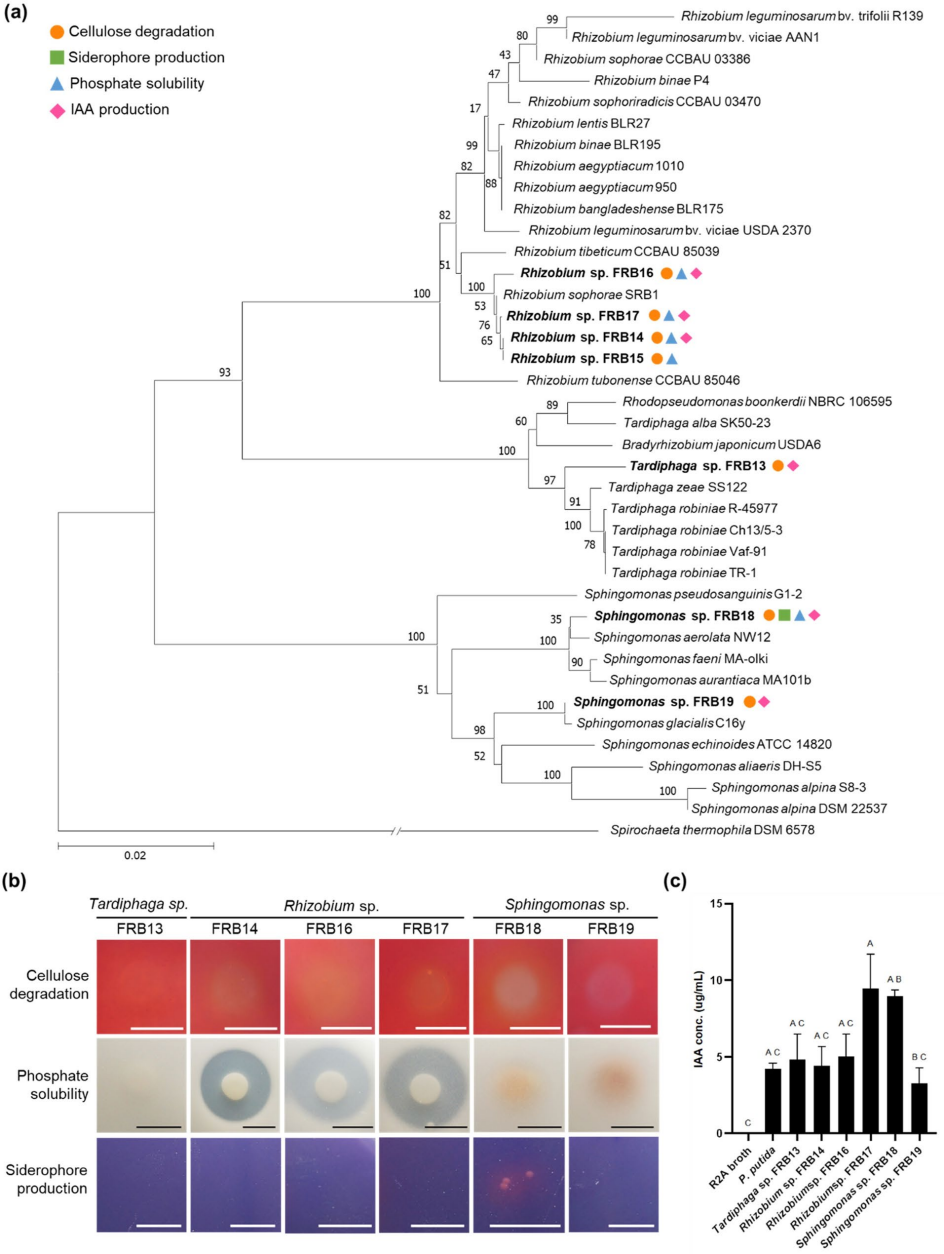
Taxonomic identity and plant growth-promoting traits of representative FRB strains.** a** Neighbor-joining phylogenetic tree based on 16S rRNA sequences of representative strains belonging to *Sphingomonas*,* Rhizobium*, and *Tardiphaga*; *Spirochaeta thermophila* was used as an outgroup. PGP related traits (cellulose degradation, phosphate solubilization, siderophore production, and IAA biosynthesis) are indicated by symbols adjacent to strain IDs. **b** Representative images showing PGP activities of six major FR strains, visualized by agar plate color change and clearing zone size/transparency (scale bar = 1 cm). **c** Quantitative comparison of IAA production in six FR strains and the reference strain *P. putida*

Four major PGP traits were evaluated by functional screening: cellulose degradation, siderophore production, phosphate solubilization and IAA production (Fig. 3). When PGP traits were confirmed in the 28 isolated strains, *Sphingomonas* spp. and *Rhizobium* spp., which were significantly more abundant in FRs, and the FRB strain belonging to *Tardiphaga* sp., which was found in only the FR samples, presented more than two PGP traits (Fig. 3a). In particular, *Sphingomonas* sp. FRB18 was found to possess all four PGP traits identified in this study.

Two *Sphingomonas* spp. isolates also presented strong multifunctional PGP traits (Figs. 3b-c). FRB19 clearly degraded cellulose, increasing the transparency of the CMC plate to 21%. FRB18, which matched ASV1, presented a transparency index of 28% and was confirmed to have stronger cellulose degradation ability than FRB19. Both strains were capable of producing IAA, with FRB18 producing higher levels of IAA than FRB19 (Fig. 3c). FRB18 produced 8.96 µg/ml IAA, which is 212% of the level produced by *P. putida* (4.23 µg/ml), a representative PGP bacterium, while FRB19 produced 3.27 µg/ml. Additionally, FRB18 exhibited weak phosphate solubility (D/d = 1.0) limited to its growth area. Although strains showed poor growth on CAS media, a distinct color change around the colonies confirmed their ability to produce siderophores (Fig. 3b, Supplementary Table S4).

Four strains belonging to genus *Rhizobium* are capable of cellulose degradation and phosphate solubilization. The D/d ratio of the *Rhizobium* spp., a parameter used to evaluate phosphate solubility, was similar at 2.5–2.9. This value was significantly greater than the average D/d ratio of 1.0 for all FRBs (Fig. 3b, Supplementary Table S4). The *Rhizobium* spp. also had the ability to produce IAA, with FRB14 and FRB16 showing the similar level of production as *P. putida* (Fig. 3c). FRB17 produced 9.48 µg/ml IAA, which is 224% of the amount by *P. putida*, demonstrating its strong ability to produce IAA.

*Tardiphaga* sp. (FRB13), identified exclusively in FR samples, has the ability to degrade cellulose and produce IAA. The FRB13 strain was confirmed to degrade cellulose by increasing the transparency of the CMC medium by 14%, and in the IAA production test, the production amount was 3.56 µg/ml, which was similar to that of *P. putida* (Fig. 3, Supplementary Table S4).

### FRBs can promote root development and growth in plants

**Fig. 4:**
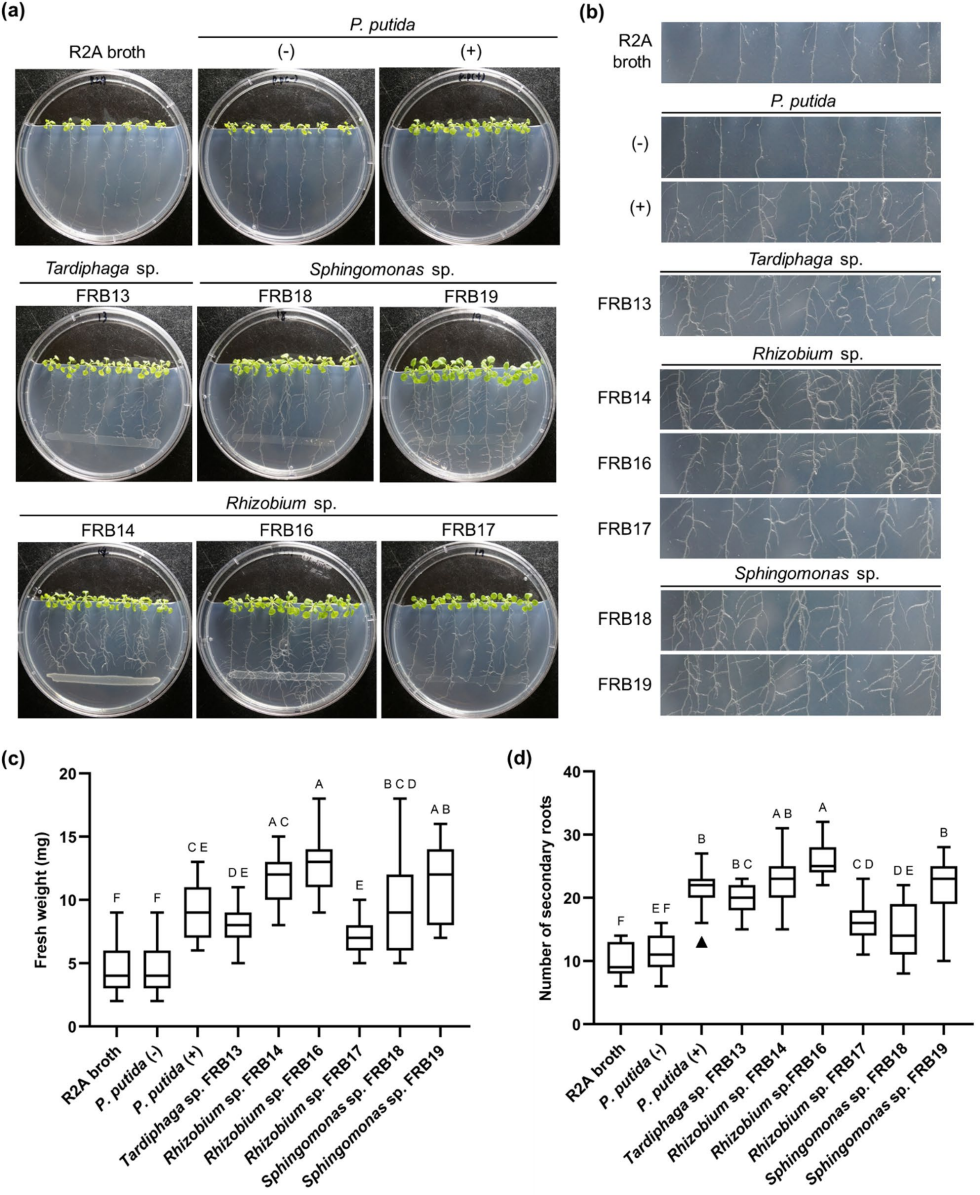
Evaluation of plant growth-promoting effects of FR bacterial strains in *A. thaliana*. **a** Representative images show *A. thaliana* seedlings grown on MS medium 11 days after inoculation with FRB strains. **b** Root development phenotypes at the same time point. Plant growth promotion was further quantified by measuring (**c**) mean fresh weight and (**d**) number of secondary roots. In all experiments, the negative controls were R2A medium alone and heat-killed *P. putida* cultures. The positive control was live *P. putida*, which is a well-characterized plant growth-promoting bacterium. All experiments were performed with a minimum of 20 plants per group (*n*> 20) in independent triplicates. Statistical analyses were conducted using one-way ANOVA, with significance at *p* < 0.05

The PGP efficacy of the FRB strains was validated in a co-culture experiment with *A. thaliana* seedlings (Fig. 4). Compared with uninoculated controls, plants inoculated with FRB strains presented larger shoots, greater fresh weights, and increased secondary root numbers (Figs. 4a-b; one-way ANOVA, *p* < 0.05). Among the *Sphingomonas* spp., FRB18 and FRB 19 increased average fresh weight by 109% and 157% (*p* < 0.0001), respectively, with FRB19 notably promoting average root branching by 131% (*p* < 0.0005). Among the *Rhizobium* spp., FRB14 and FRB16 had greater effects, with FRB16 increasing average fresh weight by 178% and the average number of secondary roots by 172% (*p* < 0.0001). FRB17 was less effective than the two previous Rhizobium spp., but it increased fresh weight by 54% (*p* < 0.05) and the number of secondary roots by 69% (*p* < 0.0001). FRB13, which has been confirmed to exist only in FRs, also increased fresh weight and root development by 78% and 102%, respectively (*p* < 0.0002).

Compared with the well-known PGPB *P. putida*, the strains FRB14, FRB16, and FRB19 presented greater PGP efficacy in terms of increasing fresh weight and secondary root production. *Sphingomonas* sp. FRB19, which corresponds ASV3, increased the fresh weight by 29% compared to *P. putida* (*p* < 0.02). Overall, the growth-promoting effects were more pronounced in *Rhizobium* spp., with FRB14 and FRB16 increasing fresh weight by 26–40% more than *P. putida* and promoting root development by 7–22% (*p* < 0.001).

## Discussion

A community shift toward particular functional groups appeared to be a general feature of FRs, regardless of the plant host [17, 52–54], and this trend also reported in other FRs. Similarly, Zotti et al. reported that FR expansion increases bacteria with the potential for mycorrhiza formation, plant growth promotion, and fungal pathogen suppression [17]. The bacterial community composition of the Antarctic moss *S. uncinata* FR revealed distinctive ecological characteristics, particularly with respect to PGPB. Our results indicate that the genera *Sphingomonas* and *Rhizobium*, which possess PGPB potential, are dominant components of the FR bacterial community, showing significant enrichment compared to healthy moss. The genus *Sphingomonas* bacteria are known to increase plant biomass by improving photosynthetic performance under environmental stress, stimulating lateral root and root hair growth, and producing gibberellins [27, 55–57]. Species of the genus *Rhizobium* are well-known PGPB that produce phytohormones and siderophores and solubilize inorganic phosphate [58, 59]. They also possess a biosynthetic pathway that produces IAA, a key regulator of root growth [59, 60]. ASV-based analysis has limitations in achieving detailed species-level classification, as seen in the case of *Rhizobium* spp., because it matches based on short ASV sequence fragments. Nevertheless, it also demonstrated the strength of this method in that, despite ASV1 and ASV3 belonging to the same genus as *Sphingomonas* spp., different strains were successfully matched, enabling a comparison of their relative abundances.

PGP traits of FR enriched bacteria identified through functional analyses can increase plant nutrient uptake by enabling phosphate solubilization, which converts insoluble phosphate into a form that plants can use, and by producing siderophores that chelate soil iron and transport it into plant cells [59, 61]. In addition, cellulase not only is known for limiting the growth of plant pathogenic fungi but also contributes to cycling soil organic matter by decomposing it into forms that plants can use as nutrients [62–64]. Like Antarctic *Pseudomonas* sp., which promote alfalfa growth *via* siderophore production [65], our data suggest that FR-associated bacteria contribute to nutrient cycling and plant development in this extreme environment.

In both the Mediterranean grassland and Antarctic *S. uncinata* FRs, *Pseudomonadota* and *Actinomycetota* were identified at higher rates than in areas with healthy plant growth. In the Mediterranean grassland, *Pseudomonadota* comprised 54.9% of the “fungal front” (FF) zone, characterized by active hyphal growth and plant mortality. However, such dominance cannot be generalized to all vegetation with FRs. In the FRs studied in other Mediterranean grasslands, the proportion of *Actinomycetota* increased to 85% in the FF zone [52]. In the FR of the montane grasslands studied in the Eastern Pyrenees, *Bacillota* dominated the equivalent fungal front zone at 53.8%, accompanied by decreases in the mean OTU proportions of *Pseudomonadota*, *Acidobacteriota*, *Actinomycetota*, and *Chlorofolx* [35]. These variations suggest that regional flora, the environment, and host identity strongly shape FR bacterial communities. Given this high variability, the findings of our study, derived from a specific coastal site, should be interpreted as a localized case study rather than a generalized model for the entire Antarctic ecosystem. Within this specific context, the dominance of *Pseudomonadota* could be a distinctive feature of the *S. uncinata* endophytic microbiota. This is consistent with previous studies that have shown similar bacterial community structures in healthy and FR-affected *S. uncinata* [34, 66, 67]. Analysis of fungal communities revealed that *Psychronectria hyperantarctica*, which dominated in the FR sample, is a species proposed as a phytopathogenic fungus inducing fairy rings in *S. uncinata* on King George Island, Antarctica [11, 13]. This suggests that the FR identified in this study also exhibits a disease phenotype caused by the same fungal strain. In the PL zone, the fungal order *Chaetothyriales* was significantly present and dominated by the genus *Cladophialophora*, which has been reported to function as a biological controller [68]. Additionally, the *Helotiales*, identified in the same location, are a well-known order of fungi associated with mycorrhizal development [69]. The increase of these potentially beneficial fungi in the PL zone, coinciding with the accumulation of mycorrhiza-helper bacteria in the adjacent FR [70], implies potential cross kingdom interactions. However, a significant proportion of the fungal community across all sample types was classified as *Incertae sedis*. This taxonomic ambiguity limits our ability to fully elucidate the precise functional roles of the fungal community and its specific interactions with the bacterial consortia. Therefore, the PGP traits reported herein are attributed specifically to the isolated bacterial strains.

We experimentally confirmed that FRB strains belonging to genera *Sphingomonas* and *Rhizobium* can promote *A. thaliana* plant growth. FRB strains that presented elevated FR-specific abundances presented even stronger PGP effects than did *P. putida*, a well-established PGPB [49, 50]. Additionally, we isolated strains that belong to the genus *Pseudomonas*, which are also widely recognized as PGPBs, and their PGP potential was confirmed in this study (Supplementary Table S4) [71–73]. However, although they were relatively easy to isolate from the *S. uncinata* FR, they did not match the ASV of bacteria present in the actual FR, and the abundance of *Pseudomonas* spp. present in tissues with the FR phenotype was only 0.004%. These results indicate that the frequent isolation of bacterial strains does not necessarily reflect their abundance in situ, underscoring the importance of integrating community-level abundance data to infer the ecological functions of specific strains.

Studies focusing on changes in the distribution and diversity of microbial communities in FRs have had limited success in analyzing increases in abundance at the genus level and the role of the associated microbes within the FR ecosystem in detail [29, 30, 34]. Furthermore, even if specific strains isolated from FRs show PGP efficacy, it is difficult to determine whether they would have a significant effect on ecological changes within FRs due to their low abundance [36]. Compared with those broader surveys, our study employed a homogeneous sampling design focused on well-developed carpets of *S. uncinata* with clearly defined health conditions, enabling high-resolution ASV-based analysis. This approach revealed clear enrichment of the genera *Sphingomonas* and *Rhizobium* in the FR samples, and their ecological relevance was further supported by the successful isolation of strains matching these ASVs. However, as the results of our pathogenicity and PGP activity tests were not obtained from mosses or vascular plants present in the Antarctic field, they should be interpreted as preliminary indicators of the role of the bacterial consortium in the Antarctic environment.

Root fungal endophytes have been suggested to play roles in succession processes in Antarctic moss FRs [34], and several fungal taxa, including endophytes, are known to promote plant growth by enhancing nutrient uptake [74–76]. Bacterial communities have sufficient capacity to facilitate positive interactions between fungal endophytes and plants. Additionally, rising temperatures in Antarctica, the expansion of plant habitats, and increased plant biomass induce changes in microbial communities, including genus *Sphingomonas* [77, 78]. Additionally, the Antarctic ecosystem is currently undergoing dynamic changes to its vegetation distribution due to climate change, human activity, and the introduction of invasive plant species [79–81]. These changes provide a basis for bacteria and fungi to play a significant role in the transition process occurring in the context of global warming. Thus, in this context, we propose the possibility that PGPBs enriched in FRs may contribute to early successional processes, although this remains a hypothesis that requires further investigation. While our study focused on bacterial communities, FRs are complex ecological systems comprising diverse fungi, bacteria, and plants. To fully understand the roles of these bacteria, future research should investigate the multifaceted interactions among bacteria, fungi, and plants in these extreme environments. Such investigations could yield valuable insights into Antarctic ecosystem dynamics and potentially inform novel biological strategies for enhancing agriculture under harsh conditions.

## Conclusion

This study identified the components and dominant groups of microbial communities present in the Antarctic *S. uncinata* FR structures through ASV sequence-based metabarcoding analysis. Additionally, dominant bacterial strains within the FRs were isolated and taxonomically identified by matching ASV sequences with 16S rRNA gene sequences of cultured isolates. The major FR bacterial strains possessed various PGP traits, with *Sphingomonas* spp. and *Rhizobium* spp. significantly enhancing the growth and root development of plants in co-culture experiments. These strains are presumed to play important roles in plant adaptation and early community establishment within the FR environment, contributing to the understanding of ecological responses of Antarctic terrestrial ecosystems to environmental changes and climate change. This study provides new insights into the microbial–plant interactions and their functional significance in extreme Antarctic environments, offering valuable implications for future ecological restoration and conservation strategies.

## Supplementary Information

Supplementary Material 1: Figure S1. Results of plant pathogenicity tests for major FRB strains. Supplementary Table S1. Summary of sequencing data for FR, PL and HL samples used in metabarcoding analysis. Supplementary Table S2. Summary of ASV processing and filtering results for FR, PL, and HL Samples. Supplementary Table S3. Accession numbers of 16S rRNA sequences used in phylogenetic analysis. Supplementary Table S4. Results of the PGP functional tests of FRB strains.

## Data Availability

The raw sequencing data have been submitted to the National Center for Biotechnology Information (NCBI) Sequence Read Archive (SRA) database under the accession number PRJNA1310811.
