# Supplementary material for: Antarctic moss fairy rings serve as reservoirs for plant growth-promoting bacteria
Source: BMC Plant Biol. 2026 Jan 20;26:299. doi: 10.1186/s12870-026-08127-3 (PMC12903237; doi:10.1186/s12870-026-08127-3)
Supplement: Supplementary file 1 — Supplementary Material 1: Figure S1. Results of plant pathogenicity tests for major FRB strains. Supplementary Table S1. Summary of sequencing data for FR, PL and HL samples used in metabarcoding analysis. Supplementary Table S2. Summary of ASV processing and filtering results for FR, PL, and HL Samples. Supplementary Table S3. Accession numbers of 16S rRNA sequences used in phylogenetic analysis. Supplementary Table S4. Results of the PGP functional tests of FRB strains. [file 12870_2026_8127_MOESM1_ESM.pdf]

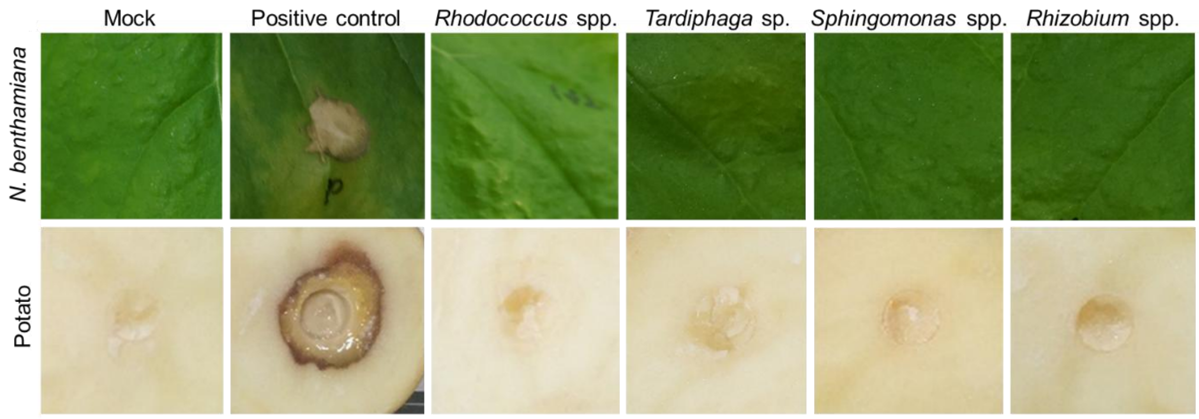

Figure S1. Results of plant pathogenicity tests for major FRB strains. Major FRB strains were selected based on ASV-based microbial community analysis results for FR, PL, and HL samples. Pathogenicity assays were performed on both leaves (N. benthamiana) and tubers (potato). In the mock condition, *N. benthamiana* leaves and potato tubers were inoculated with distilled water and 0.9% NaCl, respectively. For the positive control, *Pst* DC3000 was used on *N. benthamiana*, leaves and *P. carotovorum* was used on potato tubers. For each test, FRB strains belonging to the same genus were mixed and inoculated.

Supplementary Table S1. Summary of sequencing data for FR, PL and HL samplesused in metabarcoding analysis.

Sample name	Total read base(bp)	Total reads	GC(%)	Q20(%)	Q30(%)
FR1_16s	69,376,286	230,486	53.7	90.6	80.8
FR2_16s	59,534,790	197,790	53.8	90.2	80.5
FR3_16s	52,178,350	173,350	53.8	89.9	79.9
PL1_16s	49,387,478	164,078	53.6	90.1	80.1
PL2_16s	49,719,180	165,180	54	89.9	79.9
PL3_16s	52,839,948	175,548	54	89.3	78.9
HL1_16s	52,091,662	173,062	54.54	92.14	83.09
HL2_16s	58,268,784	193,584	54.44	92.41	83.54
HL3_16s	63,105,854	209,654	54.27	92.66	84.08
FR1_ITS	81,247,726	269,926	59.8	85.7	75.5
FR2_ITS	81,567,388	270,988	57.5	87.3	77.2
FR3_ITS	77,721,210	258,210	58.5	86.9	76.8
PL1_ITS	77,363,622	257,022	58.6	87.3	77
PL2_ITS	73,360,924	243,724	58.8	86.1	75.3
PL3_ITS	86,781,912	288,312	58	85.3	74.3
HL1_ITS	55,326,810	183,810	53.37	89.32	79.38
HL2_ITS	47,193,790	156,790	55.21	87.81	76.83
HL3_ITS	57,290,534	190,334	56.41	87.67	76.89

Supplementary Table S2. Summary of ASV processing and filtering results for FR, PL, and HL Samples

Sample name	Input reads	Filtered reads	Denoised reads	Merged reads	Non-chimeric reads
FR1_16S	115,243	96,135	92,207	60,692	47,898
FR2_16S	98,895	82,664	78,319	50,240	41,634
FR3_16S	86,675	70,925	67,813	45,867	38,739
PL1_16S	82,039	68,100	65,915	56,528	51,162
PL2_16S	82,590	67,783	64,812	50,582	45,410
PL3_16S	87,774	71,350	67,383	50,589	42,987
HL1_16s	86,531	73,904	72,139	59,153	52,903
HL2_16s	96,792	82,828	81,004	67,401	60,282
HL3_16s	104,827	91,220	89,231	77,942	72,813
FR1_ITS	134,963	113,212	112,737	106,969	100,076
FR2_ITS	135,494	115,524	114,887	110,909	98,582
FR3_ITS	129,105	109,527	109,047	106,129	98,832
PL1_ITS	128,511	107,799	107,324	106,349	99,692
PL2_ITS	121,862	100,198	99,891	97,927	91,291
PL3_ITS	144,156	114,688	114,204	111,246	100,982
HL1_ITS	91,905	75,786	75,545	75,060	73,220
HL2_ITS	78,395	61,599	61,420	60,554	59,559
HL3_ITS	95,167	76,118	75,858	74,494	72,956

Supplementary Table S3. Accession numbers of 16S rRNA sequences used in phylogenetic analysis.

Strain name	Accession number
Rhizobium leguminosarum bv. trifolii R139	OL453267.1
Rhizobium leguminosarum bv. viciae AAN1	PP047573.1
Rhizobium sophorae CCBAU 03386	NR_178709.1
Rhizobium binae P4	KX452225.1
Rhizobium sophoriradicis CCBAU 03470	NR_178708.1
Rhizobium lentis BLR27	NR_137243.1
Rhizobium binae BLR195	NR_137242.1
Rhizobium aegyptiacum 1010	NR_137399.1
Rhizobium aegyptiacum 950	JQ670253.3
Rhizobium bangladeshense BLR175	NR_137241.1
Rhizobium leguminosarum bv. viciae USDA 2370	NR_118339.1
Rhizobium tibeticum CCBAU 85039	NR_116254.1
Rhizobium sophorae SRB1	LC801609.1
Rhizobium tubonense CCBAU 85046	NR_116255.1
Rhodopseudomonas boonkerdii NBRC 106595	NR_114302.1
Bradyrhizobium japonicum USDA6	AB231927.1
Tardiphaga zeae SS122	OP556611.1
Tardiphaga robiniae R-45977	NR_117178.1
Tardiphaga robiniae Ch13/5-3	PV169197.1
Tardiphaga robiniae Vaf-91	KJ721004.1
Tardiphaga robiniae TR-1	PQ607916.1
Sphingomonas pseudosanguinis G1-2	NR_042578.1
Sphingomonas aerolata NW12	NR_042130.1
Sphingomonas faeni MA-olki	NR_042129.1
Sphingomonas aurantiaca MA101b	NR_042128.1
Sphingomonas glacialis C16y	NR_117270.1
Sphingomonas echinoides ATCC 14820	NR_024700.1
Sphingomonas aliaeris DH-S5	NR_181339.1
Sphingomonas alpina S8-3	NR_117230.1
Sphingomonas alpina DSM 22537	MT821524.1
Spirochaeta thermophila DSM 6578	NR_117123.1

Supplementary Table S4. Results of the PGP functional tests of FRB strains.

Bacteria strain	Organism	Cellulose degradation	Phosphate solubility	Siderophore production
		Transparency Index(%)	D/d	D/d
FRB1	*Rhodococcus sp.*	6.5	1.1	0.0
FRB2	*Rhodococcus sp.*	4.9	0.0	0.0
FRB3	*Mycobacterium sp.*	2.1	1.0	0.0
FRB4	*Mycolicibacterium sp.*	2.6	1.1	0.0
FRB5	*Mycolicibacterium sp.*	1.6	1.3	0.0
FRB6	*Lacisediminihabitans sp.*	14.4	1.2	0.0
FRB7	*Nakamurella sp.*	0.3	0.0	0.0
FRB8	*Mucilaginibacter sp.*	1.0	0.0	0.0
FRB9	*Priestia sp.*	29.3	1.1	0.0
FRB10	*Lichenibacterium sp.*	8.2	0.0	0.0
FRB11	*Methylobacterium sp.*	10.9	0.0	0.0
FRB12	*Methylobacterium sp.*	2.6	0.0	0.0
FRB13*	*Tardiphaga sp.*	14.1	0.0	0.0
FRB14*	*Rhizobium sp.*	22.6	2.6	0.0
FRB15	*Rhizobium sp.*	36.5	2.9	0.0
FRB16*	*Rhizobium sp.*	5.9	2.5	0.0
FRB17*	*Rhizobium sp.*	11.4	2.7	0.0
FRB18*	*Sphingomonas sp.*	28.2	1.0	3.5
FRB19*	*Sphingomonas sp.*	21.1	0.0	0.0
FRB20	*Caballeronia sp.*	1.1	0.0	0.0
FRB21	*Variovorax sp.*	43.1	0.0	0.0
FRB22	*Rugamonas sp.*	22.1	0.0	0.0
FRB23	*Pseudomonas sp.*	49.5	2.2	3.0
FRB24	*Pseudomonas sp.*	32.8	2.6	4.0
FRB27	*Pseudomonas sp.*	15.4	0.0	2.9
FRB29	*Pseudomonas sp.*	31.1	1.3	2.3
FRB32	*Rhodanobacter sp.*	70.9	3.5	0.0
FRB33	*Acidiphilium sp.*	0.6	0.0	0.0

*The strains used in the plant growth promotion test using plant co-culture assays.
